# Hierarchical Modular Structure Identification with Its Applications in Gene Coexpression Networks

**DOI:** 10.1100/2012/523706

**Published:** 2012-12-30

**Authors:** Shuqin Zhang

**Affiliations:** Center for Computational Systems Biology, School of Mathematical Sciences, Fudan University, Shanghai 200433, China

## Abstract

Network module (community) structure has been a hot research topic in recent years. Many methods have been proposed for module detection and identification. Hierarchical structure of modules is shown to exist in many networks such as biological networks and social networks. Compared to the partitional module identification methods, less research is done on the inference of hierarchical modular structure. In this paper, we propose a method for constructing the hierarchical modular structure based on the stochastic block model. Statistical tests are applied to test the hierarchical relations between different modules. We give both artificial networks and real data examples to illustrate the performance of our approach. Application of the proposed method to yeast gene coexpression network shows that it does have a hierarchical modular structure with the modules on different levels corresponding to different gene functions.

## 1. Introduction

 Networks are widely applied to model complex systems, including biological systems, social organizations, World-Wide-Webs, and so on. In a network, the nodes (vertices) represent the members in the system, while the edges represent the interactions among the members. If two nodes have interactions in a network, there will be an edge connecting them. With such a representation, the complex systems can be analyzed by computational methods.

Module (community) structure is a common property of many different types of networks. Modules are the dense subgroups of a network, where the nodes in the same module are more likely to connect each other than the nodes in other modules. In general, the members in the same module share some common properties or play similar roles. For example, in a gene coexpression network, the genes in the same module may belong to the same functional category such as lipid metabolism and acute-phase response [1]. Since the paper published by [2], module detection and identification becomes a hot research topic in several different areas such as computer science, physics, and statistics. A large number of related works have been published with the physicists making the most contributions [3–12]. Several recent review papers provide details and comparisons of the module identification methods [6, 9, 13]. Reference [13] compares the performance of several existing methods for both computation time and output. Reference0020[6] is a thorough, more recent discussion. Reference [9] contrasts different perspectives of the methods and sheds light on some important similarities of several methods. A recent comparison of some popular methods is shown in [14]. Among the compared methods, the method by maximizing the average degree within modules and minimizing the average connections between different modules outperforms other methods in identification accuracy. Its computational speed is also competitive [14]. Besides these computational methods, theoretical analysis on module identifications is presented very recently. Bickel and Chen gave the first statistical analysis on the properties of modules [15]. There based on the stochastic block model, they gave the sufficient conditions for a modularity to be a consistent estimator of modules and presented a new consistent modularity. However, the computation of maximizing this modularity is very time consuming.

**Figure 1:**
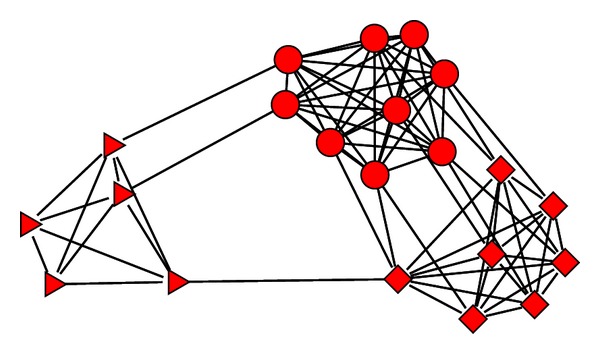
Example of hierarchical modular network structure.

Although so many related works are published, how to choose an appropriate number of modules keeps being an open problem. Different methods output different solutions when they are applied to the same network. In reality, all of the different choices may be reasonable because different choices of this number may correspond to the modules on different levels. As explained in [16], some modular networks may have hierarchical structure. For example, in a friendship network, on the large scale, the modules may correspond to people from different countries. On the smaller scales, people in the same module may graduate from the same university, grow up in the same community, or even be born in the same family. Such hierarchical modular structure appears in different kinds of networks. For example, Meunier and colleagues gave an example on hierarchical modular structures in human brains [17].  Figure 1 shows an example of hierarchical modular network. There are two levels of the modules. We can identify three modules corresponding to different shapes of nodes on the lowest level or two modules with nodes represented by cubes and circles being combined together on the higher level. 

 Compared to the module identification in a partitional way (all the modules are on the same level), there are much fewer works on computational methods for hierarchical modular structure analysis [18–20]. Although these papers present some methods to construct the hierarchical modular structure, they do not give a clear picture on how these modules are organized and what the relationship among the modules is. In this paper, we mainly consider the problem of hierarchical modular structure in unweighted networks. Based on the module identification method presented in [14], we give the method on how to construct the hierarchical structure from all the possible modules in Section 2. Numerical experiments for both simulated networks and real data networks are presented to show the performance of our proposed method in Section 3. The application of the proposed method to yeast gene coexpression network shows that it does have a hierarchical structure, which corresponds to the different levels of gene functions. Conclusion remarks are given finally. By constructing the hierarchical structure, we aim to explore the functions of modules on different levels and explain why the number of modules may differ for different identification methods.

## 2. Methodology

 Before going to the details on how to construct the hierarchical structure, we give its definition first. We consider a network *G*(*V*, *E*) with *n* nodes, where *V* denotes the set of nodes and *E* denotes the set of edges. The adjacency matrix is denoted as *A* with each entry being 0 or 1. The hierarchical structure of a network is defined based on the stochastic block model, which is a direct extension of the Erdös-Rényi random graph model [21]. The network is obtained by starting with a set of *n* nodes and adding edges between them in a probabilistic fashion. The presence of an edge between any two nodes is a Bernoulli event where the probability may be vertex-pair dependent. At the beginning, we assume there are *K* modules in the network. The network is generated in two steps. First, any node is assigned to a module *M*_*i*_ with a probability *μ*_*i*_, where *μ* = (*μ*_1_, *μ*_2_,…, *μ*_*K*_) satisfies ∑_*i*=1_^*K*^ *μ*_*i*_ = 1. Then any two nodes *u*, *v* ∈ *V* and *u* ∈ *M*_*i*_, *v* ∈ *M*_*j*_ are connected with probability *P*_*i*,*j*_ depending on *M*_*i*_, *M*_*j*_, and *P* is symmetric. If there is the modular structure in the network, then *P*_*i*,*j*_ < min⁡{*P*_*i*,*i*_, *P*_*j*,*j*_}. With this model, the hierarchical structure of a network can be defined recursively. For any three modules *M*_*i*_, *M*_*j*_, and *M*_*k*_, if *P*_*i*,*j*_ > max⁡{*P*_*i*,*k*_, *P*_*j*,*k*_}, we say there is hierarchical structure among these three modules and *M*_*i*_, *M*_*j*_ can be combined to a new module parallel to *M*_*k*_.

To construct the hierarchical structure, we use the bottom-up strategy. We first find all the possible modules on the lowest level and then build the hierarchical structure. We use the method presented in [14] to find all the possible modules. Suppose *K* is given first. We let *N*_*k*_ denote the number of nodes in subnetwork *V*_*k*_, *L*_*kk*_ denote twice the total number of edges in subnetwork *V*_*k*_, and *L*_*kl*_ denote the total number of connections between the subnetworks *V*_*k*_ and *V*_*l*_, where *k*, *l* = 1,2,…, *K*. The module identification problem is formulated as

(1) max⁡ PΦ(P)=∑k=1KLkkNk−∑k=1K ∑l≠kLklNk,

where **P** is a partition of the network.

In matrix form, if we let

(2) Sik={1,if  node  i∈Vk0,otherwise i=1,2,…,n,

the problem is formulated as

(3) max⁡Ψ(S)=∑k=1KS·,kTAS·,kS·,kTS·,k−∑k=1K ‍∑l≠kS·,kTAS·,lS·,kTS·,k      =∑k=1KS·,kT(2A−D)S·,kS·,kTS·,ks.t. Si,j∈{0,1} for  i,j=1,2,…,K,    ∑k=1KS·,k=1.

Here 1 is a vector with all elements being 1.

The objective function aims to both maximize the average degree within each module and minimize the average connections between different modules. We expect to achieve a good balance of the module size and make correct inference on the modules. The problem (3) is solved with an approximate method similar to the spectral clustering. We first compute the *K* eigenvectors of the matrix 2*A* − *D*. By clustering these *K* eigenvectors as a matrix of *n* objects with *K* dimensions, we get the assignment of the *n* nodes into *K* modules.

Now, we discuss how to determine the lowest level of all the possible modules *K*. For any node *i* ∈ *V*, the degree can be written as

(4) di=∑k=1Kdi(Vk),

where

(5) di(Vk)=∑j∈VkAij,

which defines the connections that node *i* has in the subnetwork *V*_*k*_. To determine the number of possible modules, we compare the average connectivity within a subnetwork and the average connectivity between it and any other subnetwork. If the average connectivity within a subnetwork is greater, we take it as a module, that is,

(6) ∑i∈Vkdi(Vk)Nk>∑i∈Vkdi(Vl)Nk, l≠k.

Alternatively, it can also be written as

(7) Lkk>Lkl,

if we multiply both sides with *N*_*k*_. This condition is very weak, thus with it, we hope we find all the modules as on the lowest level. We do the clustering for *K* increasing from two until the condition (6) does not hold and get all the possible modules. The efficiency of the above algorithm can be seen in [14].

Based on the above results, we construct the hierarchical structure in an agglomerative way (bottom-to-up). We directly use connection probability, which is computed from the clustering results through maximum likelihood estimation, to measure the distance between different modules. This connection probability matrix is denoted as ${\hat{P}}^{0}$. First the maximum connection probability between different modules is found, and we assume it is ${\hat{P}}_{i_{0},j_{0}}^{0}$ with the corresponding two modules *i*_0_, *j*_0_ being recorded. The second largest connection probability for these two modules *i*_0_, *j*_0_ are also found, and we assume they are ${\hat{P}}_{i_{0},k_{0}}^{0}$ and ${\hat{P}}_{j_{0},l_{0}}^{0}$ with the corresponding modules being *k*_0_ and *l*_0_. To determine whether there is a hierarchical structure for these modules, we use Fisher exact test to see whether the connection probabilities ${\hat{P}}_{i_{0},k_{0}}^{0}$ and ${\hat{P}}_{j_{0},l_{0}}^{0}$ are the same as ${\hat{P}}_{i_{0},j_{0}}^{0}$. That is, we need to test ${\hat{P}}_{i_{0},j_{0}}^{0}={\hat{P}}_{i_{0},k_{0}}^{0}$ and ${\hat{P}}_{i_{0},j_{0}}^{0}={\hat{P}}_{j_{0},l_{0}}^{0}$. Here we take a *P* value threshold to be 0.05. Three different cases may occur for these two relations. (1) Both of these two null hypotheses are rejected. In this case, there is hierarchical structure and the modules *i*_0_, *j*_0_ are on the lower level than *k*_0_ and *l*_0_. We combine the two modules *i*_0_ and *j*_0_ and take them as one module. (2) Only one of ${\hat{P}}_{i_{0},j_{0}}^{0}={\hat{P}}_{i_{0},k_{0}}^{0}$ and ${\hat{P}}_{i_{0},j_{0}}^{0}={\hat{P}}_{j_{0},l_{0}}^{0}$ is accepted. The corresponding modules having the same connection probability are combined together. We look for the next largest connection probability for these three modules and test the relationship again. If two modules are tested to have the same connection probability, they are combined into one group, and the same step is implemented again. (3) Both of these two null hypotheses are accepted. These modules are taken as on the same level and combine together. We search the next largest connection probability to these four modules and do the statistical test until the hierarchical structure occurs or all the modules are combined together. After the above steps are finished, the connection probability between different modules is recalculated and recorded as ${\hat{P}}^{1}$. The above search and test steps are repeated for ${\hat{P}}^{1}$. Such steps are implemented recursively until all the modules are combined into one big module/network. For the statistical tests, we can also use *t*-test to test the relations between the connection probabilities if the distribution of the connections between different modules can be approximated by normal distribution. With this method, we can efficiently combine the modules with the same connection probability into the same level.

## 3. Numerical Experiments

 In this section, we evaluate the performance of our proposed method through its application to several examples. We first start with two artificial networks having comparatively clear module structures. We then apply our method to two real networks to evaluate its performance. The first real network is the well-known karate club network and the second one is a yeast gene coexpression network. 

### 3.1. Artificial Networks 

#### 3.1.1. A Network Composed of Cliques

 We consider a network with 200 nodes, which is composed of 4 cliques. The sizes of the cliques are 90, 30, 40, and 40. The connections between different cliques are randomly generated with the following probability:

(8) P=(1.0000.2000.0020.0030.2001.0000.0050.0100.0020.0051.0000.0300.0030.0100.0301.000).

**Figure 2:**
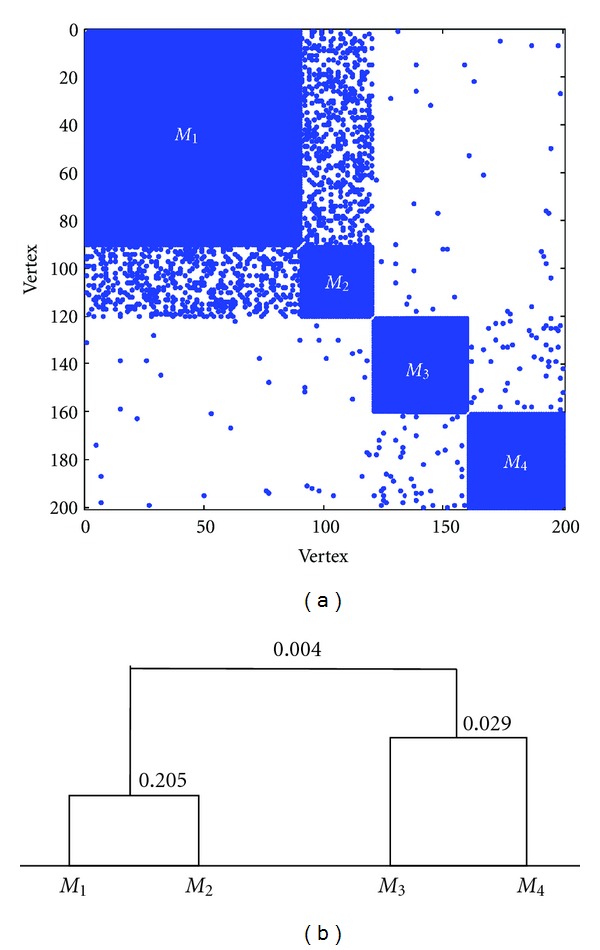
Example of hierarchical modular network structure. (a) Pattern of the adjacency matrix; (b) the hierarchical structure of the network.

The pattern of the adjacency matrix is shown in Figure 2(a). From upper-left to lower-right, we denote the four modules as *M*_1_, *M*_2_, *M*_3_, and *M*_4_, which correspond to the position in the connection probability matrix. We can see the hierarchical structure of the network from the adjacency matrix. We apply our proposed method to this network. The condition (6) is satisfied until *K* = 4. The estimated connection probability matrix is

(9) P^=(1.0000.2050.0030.0030.2051.0000.0060.0090.0030.0061.0000.0290.0030.0090.0291.000).

We apply statistical tests to the corresponding modules, and finally we get the hierarchical structure as shown in Figure 2(b). The values on the hierarchical tree is the estimated connection probability of the corresponding modules. On the lowest level, there are four modules. If the tree is cut between 0.205 and 0.029, there are three modules while if the cutoff is greater than 0.029, there are only two modules. These results are consistent with the network generation strategy.

#### 3.1.2. A Randomly Generated Network

**Figure 3:**
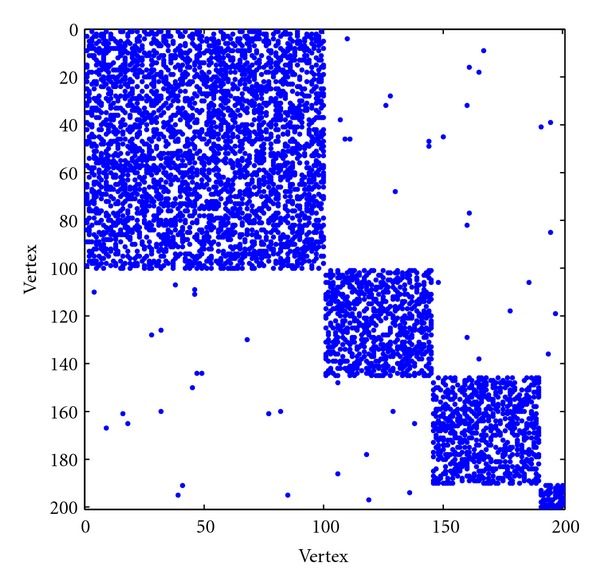
Pattern of the adjacency matrix for the randomly generated network.

 In this example, we also consider a network with 200 nodes and 4 modules. The size of each module is 10, 45, 45, and 100. We set the degree of each node within its module to be 6, 15, 15, and 30. Then the connections between different nodes are randomly generated. We keep all the edges generated for each node. So finally the average degree within each module is greater than the prespecified number. The connection probability between different modules is 0.002. The pattern of the adjacency matrix is shown in Figure 3. From upper-left to lower-right, the four modules are *M*_1_, *M*_2_, *M*_3_, and *M*_4_, respectively. With our proposed method, the network is partitioned into four modules correctly on the lowest level and the estimated connection probability is

(10) P^=(0.2980.0020.0020.0030.0020.3280.0020.0040.0020.0020.3210.0000.0030.0040.0000.560).

By using the statistical tests, these four modules are determined as parallel modules, which is the same as that in our network generation strategy.

### 3.2. Karate Club Network

**Figure 4:**
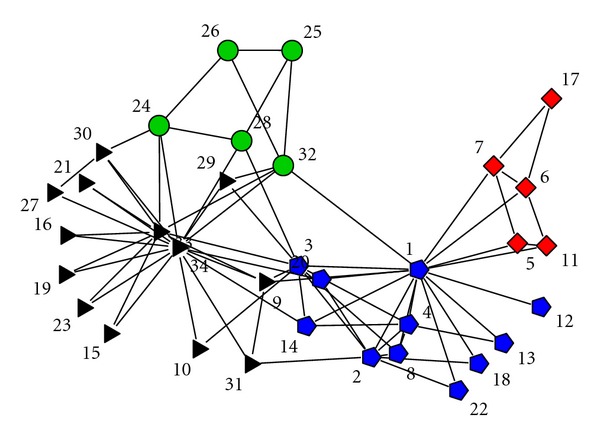
Zachary's karate club network. Different shapes show the modules. *M*_1_: pentagon, *M*_2_: square, *M*_3_: triangle, *M*_4_: circle.

 We consider the Zachary's network of karate club members [22] in this example. There are 34 nodes in this network corresponding to the members in a karate club. This dataset has been applied as a benchmark to test many module identification algorithms since the true modules are known in this network. The people in the club were observed for a period of three years. The edges represent connections of the individuals outside the activities of the club. At some point, the administrator and the instructor of the club broke up due to a conflict between them. The club was separated into two groups supporting the administrator and the instructor.  Figure 4 shows the network. Originally, there are two modules, which have 16 nodes (squares and pentagons in the figure) and 18 nodes (circles and triangles in the figure), respectively.

We apply our proposed method to this network. The criterion (6) is satisfied until *K* = 4. The result is shown in Figure 4, with different shapes of the nodes denoting different modules. The estimated connection probability matrix is

(11) P^=(0.3640.0730.0560.0360.0730.4800.0000.0000.0560.0000.2370.1080.0360.0000.1080.480).

From this matrix, it is easy to see that *M*_3_ and *M*_4_ are more likely to connect each other. With statistical tests, we can get that the connection probability among *M*_3_, *M*_4_, and *M*_1_ is the same. Although *M*_2_ has no connections to *M*_3_ and *M*_4_, it has a larger connection probability to *M*_1_ than *M*_3_, *M*_4_ to *M*_1_. Thus these four modules are on the same level. In [19], the authors considered constructing the hierarchical modular structure of this network too. At first, they also found four modules on the lowest level. Then they found that this network has two modules with some nodes (3, 9, 10, 14, 31) belonging to both of them. We did not consider the overlapping nodes in this article. However, we can see that because these overlapping nodes belong to both *M*_1_ and *M*_3_, and they connect both parts closely, our method detect *M*_1_ and *M*_3_, *M*_3_ and *M*_4_ as having the same connectivity.

### 3.3. Hierarchical Modular Structure in Yeast Gene Coexpression Network

In this section, we apply our proposed approach to analyze a gene coexpression network of yeast. The data set we use was generated by Brem and Kruglyak from a cross between two distinct isogenic strains BY and RM [23]. As described in [23], a total of 5740 ORFs were obtained after data preprocessing. In our analysis, we only use the 1,800 most differentially expressed genes as input to construct coexpression network and derive modules. When constructing the adjacency matrix of the network, we use the hard thresholding, that is: if the absolute value of Pearson correlation coefficient between two genes is greater than some given value, we assign an edge between them; otherwise, there is no edge. We compute the linear regression coefficient between the frequency of degree *d* (log⁡10(*f*(*d*))) and the log⁡10 transformed degree *d* (log⁡10(*d*)), and choose the threshold that leads to approximately scale free property of the network as described in [24]. Finally, the threshold is set to be 0.705, $\hat{R}$ is about 0.75. By such a setting, this gene coexpression network is divided into 690 unconnected parts with the largest part of size 788. Here, we only analyze the hierarchical modular structure of the largest connected network. 

**Figure 5:**
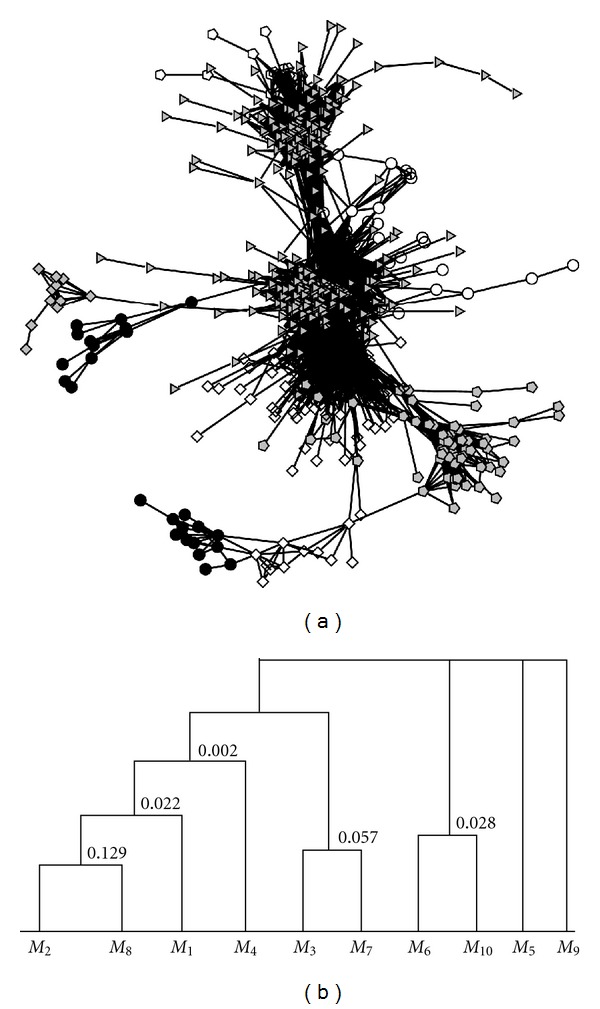
Yeast gene coexpression network. (a) The network structure, (b) the hierarchical structure.

Starting from *K* = 2, we apply the method in [14] to this network, and the condition (6) holds until *K* = 10. To make the solution more accurate, we do a global maximization by changing the module index of boundary nodes starting from the approximate solution. Since the approximate solution is already good, this step is very fast. The structure of the network is shown in Figure 5(a), with different colors and shapes denoting different modules as described in Table 1. Then we construct the hierarchical modular structure as shown in Figure 5(b). On the lowest level, there are ten modules, while on the highest level, there are four modules.

Since coexpressed genes tend to be coregulated and possibly have similar functions, genes in the same module are expected to be enriched for some function categories. In order to understand the biological basis of the network modules, we consider each identified module for enrichment of annotations from gene ontology (GO) [25]. In our analysis, the enrichment analysis was performed by GO stats from Bioconductor. For each module, the statistically most significant GO categories are analyzed. Table 1 shows the enrichment results for the ten modules. “M-size” and “G-size” are the size of both the modules and the GO categories, respectively. “Overlap” is the overlap size of the module and the GO category.  Table 2 shows the enrichment results for the modules on different levels. From the tables, it is easy to see that different gene function categories are enriched most on different levels. For example, module *M*_2_ enriches the GO category “translation" most significantly, while the combined module *M*_2_, *M*_8_ enriches “Ribonucleoprotein complex biogenesis" most significantly, with *M*_2_ containing 42 genes having this function. The combined module *M*_2_, *M*_8_, *M*_4_, and *M*_1_ also enriches this function, while *M*_4_ itself enriches “cellular respiration" significantly. On the uppermost level, the module composed of *M*_2_, *M*_8_, *M*_1_, *M*_4_, *M*_3_, and *M*_7_ enriches four GO function categories most significantly, and all the genes are overlapped. Three (“cellular component biogenesis,” “cellular component biogenesis at cellular level,” and “ribosome biogenesis”) of them are different from the most enriched gene functions for each of these six modules. All these results indicate that hierarchical modular structure does exist in gene coexpression networks and different gene functions are enriched most on different levels.

**Figure 6:**
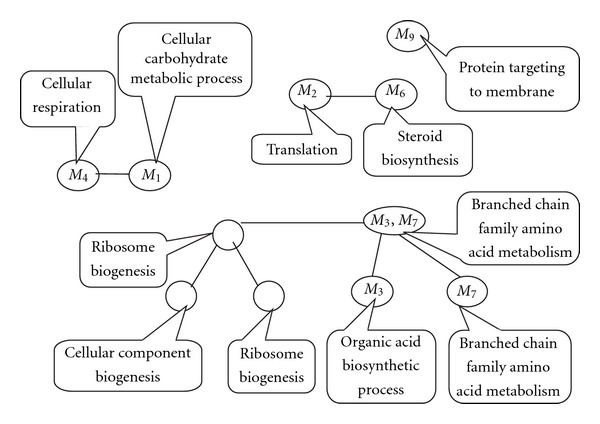
Tree map of the enriched GO categories in yeast gene coexpression network.

We use the software REViGO to check the hierarchical structure of the enriched GO categories [26]. We consider the enriched GO categories in Tables 1 and 2 except the category “regulation of translational termination" because its G-size is very small and the *P* value is comparatively large.  Figure 6 shows the tree map of the most enriched GO categories. The subgraph that we do not mark with the module corresponds to the combined module *M*_1_, *M*_2_, *M*_3_, *M*_4_, *M*_7_, *M*_8_. Here the modules *M*_6_, *M*_9_ and other modules are parallel to each other, which is consistent with our results. *M*_3_ and *M*_7_ belong to a large category, which is “branched chain family amino acid metabolic process". This large category is different from the most enriched category for the combined module *M*_3_ and *M*_7_. This may come from the fact that since *M*_7_ is very small, it does not cover a large part of its enriched category. *M*_1_ and *M*_4_ are parallel to each other which is also consistent with our analysis. All these results show that our proposed method can explain some of the hierarchical structure of the GO categories. Due to the network size, we did not handle all the genes of yeast. This may be a reason why some of our computational results are not consistent with the GO function tree map.

## 4. Conclusion

Module identification problem has attracted much attention from different fields and it continues being a hot research topic. How to determine the number of modules in a modular network has been an open problem during the study of module identification methods. This problem may come from the hierarchical structure of modular networks. The different numbers correspond to the different levels of the hierarchical structure and they may be all reasonable. In this paper, we proposed a method for constructing the hierarchical modular structure of networks. With statistical tests, we can identify both the parallel modules and the hierarchical structure. According to different cutoffs of the hierarchical tree, different numbers of modules can be identified. This may solve the problem of the number of network modules to some extent. Several examples are given to demonstrate the efficiency of our method. Application of this method to gene coexpression networks shows that there are hierarchical modules in yeast gene coexpression network. On different levels of such networks, the genes in the module belong to different gene functions most. Thus studying the gene function through constructing the hierarchical modular structure instead of specifying the number of modules should perform better. Application of such algorithms to other kinds of networks may also contribute to other research fields.

## Figures and Tables

**Table 1:** GO enrichment analysis results of the gene modules on the lowest level.

Module	Color, shape	*M*-size	Enriched GO category	*P* value	*G*-size	Overlap
*M*_1_	White, square	190	Cellular carbohydrate metabolic process	3.23 × 10^−9^	60	35
*M*_2_	White, circle	126	Translation	4.70 × 10^−59^	101	80
*M*_3_	Grey, triangle	135	Organic acid biosynthetic process	5.41 × 10^−35^	89	64
*M*_4_	Grey, pentagon	62	Cellular respiration	4.13 × 10^−27^	36	28
*M*_5_	Black, circle	12	Amino acid catabolic process to alcohol via Ehrlich pathway	1.76 × 10^−7^	5	4
*M*_6_	Black, circle	13	Steroid biosynthetic process	2.20 × 10^−15^	13	9
*M*_7_	White, pentagon	19	Branched chain family amino acid metabolic process	4.37 × 10^−8^	11	6
*M*_8_	Grey, triangle	209	Ribonucleoprotein complex biogenesis	5.94 × 10^−39^	149	106
*M*_9_	Grey, square	11	Protein targeting to membrane	8.91 × 10^−6^	4	3
*M*_10_	White, square	11	Regulation of translational termination	1.55 × 10^−4^	2	2

**Table 2:** GO enrichment analysis results of gene modules on the upper level.

Module	*M*-size	Enriched GO category	*P* value	*G*-size	Overlap
*M*_2_, *M*_8_	335	Ribonucleoprotein complex biogenesis	4.02 × 10^−66^	149	148
*M*_1_, *M*_2_, *M*_8_	525	Ribonucleoprotein complex biogenesis	1.33 × 10^−29^	149	148
*M*_1_, *M*_2_, *M*_4_, *M*_8_	587	Ribonucleoprotein complex biogenesis	6.04 × 10^−23^	149	149
*M*_3_, *M*_7_	154	Organic acid biosynthetic process	9.22 × 10^−40^	89	71
*M*_1_, *M*_2_, *M*_3_, *M*_4_	741	Cellular component biogenesis	4.01 × 10^−6^	175	175
*M*_7_, *M*_8_		Cellular component biogenesis at cellular level	1.84 × 10^−5^	156	156
		Ribonucleoprotein complex biogenesis	3.19 × 10^−5^	149	149
		Ribosome biogenesis	3.44 × 10^−5^	148	148
*M*_6_, *M*_10_	24	Steroid biosynthetic process	2.36 × 10^−19^	13	12
